# Geographical variation in hepatitis C-related severe liver disease and patient risk factors: a multicentre cross-sectional study

**DOI:** 10.1017/S0950268823000377

**Published:** 2023-03-14

**Authors:** Sema Nickbakhsh, E. Carol McWilliam Leitch, Shanley Smith, Chris Davis, Sharon Hutchinson, William L. Irving, John McLauchlan, Emma C. Thomson

**Affiliations:** 1 MRC-University of Glasgow Centre for Virus Research, College of Medical, Veterinary and Life Sciences, University of Glasgow, Glasgow, G61 1QH, UK; 2 Public Health Scotland, Meridian Court, 5 Cadogan Street, Glasgow G2 6QE, UK; 3 NIHR Nottingham Digestive Diseases Biomedical Research Unit, University of Nottingham, Nottingham NG7 2RD, UK

**Keywords:** Cirrhosis, public health, risk maps, viral hepatitis

## Abstract

Despite promising steps towards the elimination of hepatitis C virus (HCV) in the UK, several indicators provide a cause for concern for future disease burden. We aimed to improve understanding of geographical variation in HCV-related severe liver disease and historic risk factor prevalence among clinic attendees in England and Scotland. We used metadata from 3829 HCV-positive patients consecutively enrolled into HCV Research UK from 48 hospital centres in England and Scotland during 2012–2014. Employing mixed-effects statistical modelling, several independent risk factors were identified: age 46–59 y (OR_adj_ 3.06) and ≥60 y (OR_adj_ 5.64) relative to <46 y, male relative to female sex (OR_adj_ 1.58), high BMI (OR_adj_ 1.73) and obesity (OR_adj_ 2.81) relative to normal BMI, diabetes relative to no diabetes (OR_adj_ 2.75), infection with HCV genotype (GT)-3 relative to GT-1 (OR_adj_ 1.75), route of infection through blood products relative to injecting drug use (OR_adj_ 1.40), and lower odds were associated with black ethnicity (OR_adj_ 0.31) relative to white ethnicity. A small proportion of unexplained variation was attributed to differences between hospital centres and local health authorities. Our study provides a baseline measure of historic risk factor prevalence and potential geographical variation in healthcare provision, to support ongoing monitoring of HCV-related disease burden and the design of risk prevention measures.

## Introduction

The latter part of the 20th century witnessed an alarming growth in liver disease deaths in the UK, with an estimated 250% increase between 1971 and 2001 [1]. Viral hepatitis is a major cause of liver disease and liver transplantation, on a par with excessive alcohol consumption and obesity [1]. However, despite a rise in hepatitis C-related end-stage liver disease and hepatocellular carcinoma in the early 2000s [2], new direct acting antiviral (DAA) treatments have reversed this trend in many countries including the UK. The estimated numbers of patients now living with chronic hepatitis C virus (HCV) infection has diminished in England from around 129 000 people in 2015 to 81 000 in 2020 [3], and in Scotland from 1814 in 2015 to 1423 in 2018 [4]. Furthermore, recent Scottish estimates of chronic hepatitis C prevalence among people who inject drugs (PWID) has shown a decrease from 37% in 2015–16 to 19% in 2019–20 [5].

However, most diagnosed cases of HCV infection remain untreated globally [6]. Furthermore, despite promising steps towards the elimination of HCV in the UK, evidence suggests the incidence of new HCV infections may not be in decline, and the COVID-19 epidemic has impacted significantly on access to treatments and harm prevention services [3, 7–9] threatening the UKs ability to meet the WHO 2030 target of eliminating viral hepatitis [10]. Targeted risk prevention interventions therefore remain an important adjunct to early diagnosis and treatment in controlling the burden of hepatitis C.

The prevalence of hepatitis C, and HCV-related healthcare access and usage, is known to vary geographically in the UK owing in part to levels of social deprivation and provision of healthcare [1]. However, geographical variation in HCV-related liver disease and associated patient correlates are not well understood. Improved knowledge may enable an individualised approach to risk factor reduction interventions at a local level. Several demographic, behavioural and clinical factors are known to contribute to the risk of progression to severe stages of liver disease in patients with HCV infection, which may act individually or in tandem with other major risk factors [11–13]. However the role of some factors, such as HCV-genotype, remain controversial [12, 14, 15].

Population-level data are routinely collected in the UK to inform on geographical patterns in the burden of HCV-associated end-stage liver disease and mortality. However, data characterising the wider underlying population living with HCV-associated cirrhosis are lacking. These data have importance for quantifying risk factors for cirrhosis as a marker for severe disease, to target interventions and prevent serious outcomes at a local level. Here we exploited the breadth of geographical coverage and patient metadata collated through HCV Research UK (HCVRUK) between 2012 and 2014 in a cross-sectional study.

Our study focuses on the pre-DAA era, enabling improved insights into the pathogenesis of severe liver disease (as represented by cirrhosis) and providing baseline metrics to support the ongoing monitoring of trends in HCV-related severe liver disease. We aimed to: (i) assess variation across local health authorities in the proportion of severe liver disease and risk factor prevalence among patients attending specialist HCV clinics in England and Scotland; (ii) identify demographic, social and clinical risk factors associated with severe liver disease among clinic-attendees and (iii) investigate how much of the geographical variation in severe disease may be explained by patient risk factors.

## Methods

### HCVRUK and study design

HCVRUK was initiated in 2012 to provide the first nationwide HCV Clinical Database and Biobank [16]. For the study period, spanning 8th March 2012 to 30th September 2014, 4048 patients with chronic HCV infection were consecutively enrolled during routine attendance at 48 HCV specialist hospital centres, or through provision of secondary care at associated community clinics, located in England (*n* = 39) and Scotland (*n* = 9). During this period, all patients attending liver services were approached for recruitment (we note a subsequent phase of HCVRUK involved enhanced enrolment of cirrhotic patients; these participants were not included). A small proportion (25.9%; *n* = 992) of patients were recruited at one of seven adult liver transplant services.

Most recruited patients (77%; *n* = 3129) were clinic re-attenders. Of the remaining patients, most were new clinic referrals (21%; *n* = 855), the majority of whom had not previously received treatment (98% compared to 64% of re-attenders). A small number were clinically discharged treated resolvers (*n* = 2; 0.05%), and one patient was a clinically discharged spontaneous resolver (*n* = 1; 0.02%). Most patients (88%; *n* = 3568) were recruited at the hospital site, whilst a small proportion (10%; *n* = 419) were recruited through community clinics providing secondary care. A small number had incomplete information around the recruitment process (*n* = 61; 1.5%).

**Fig. 1.**
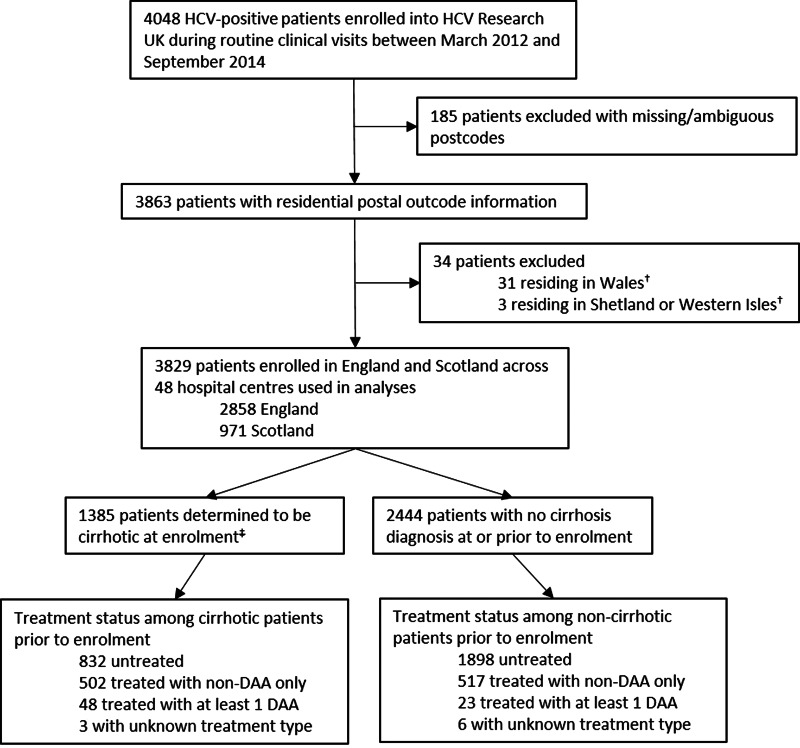
Outline of study profile. ^†^Excluded due to small numbers. ^‡^Patients with cirrhosis diagnosed at any stage during the 31-month study period were assumed to be cirrhotic at enrolment.

All study patients had a previous laboratory confirmed diagnosis of HCV infection. Data collated at enrolment and historical clinical information were analysed in a cross-sectional design, with each patient having a single associated record. Figure 1 summarises patient exclusions; 185 with missing/erroneous postcodes, and 34 from Wales, Shetland or the Western Isles due to small patient numbers, leaving 3829 patient records. These exclusions did not alter the distribution of reasons for clinic attendance or the proportions treated among these groups. The Scottish HCV Clinical database (source Public Health Scotland) was inspected to assess the representation of severe liver disease prevalence by the study population.

### Health outcomes

Stage of liver disease was recorded based on physician-diagnosed clinical evidence of cirrhosis and Ishak scoring of fibrosis following liver biopsy. Fibroscan scores were not recorded. Two groups were formed: severe liver disease (advanced fibrosis or cirrhosis; Ishak scores 4–5) and mild liver disease (without advanced fibrosis or cirrhosis; Ishak scores 0–3). Patients with cirrhosis diagnosed at any stage during the 31-month study period were assumed to be cirrhotic at enrolment. Those diagnosed with decompensation, hepatocellular carcinoma, or those undergoing liver transplantation at any time point from enrolment, were also deemed as having severe liver disease at enrolment.

### Patient risk factors for severe liver disease

Each patient completed a questionnaire with the study nurse covering demographic and current or historic social and behavioural factors: age, sex, ethnicity, country of birth, history of injection drug use (IDU) use, probable route of infection, alcohol consumption and body mass index (BMI). Clinical data was also accrued from patient health records including information on diabetes, HCV genotype and HIV-coinfection.

### Geographical data

Office for National Statistics (ONS) Postcode Directory data (November 2018) for the UK [17] were used to assign Local Health Authority (LHA) areas of residence for each patient (Regional Health Authority's for England and NHS Boards for Scotland).

### Statistical analysis

The proportion of study patients with severe liver disease and associated risk factors were generated for each LHA area. The Coefficient of Variation (CV) and Coefficient of Quartile Variation (CQV) were estimated to assess geographical variation with 95% confidence intervals generated using normal approximation. An asymptotic test for the equality of CV was conducted comparing each coefficient with a hypothesised population assuming an equivalent mean but zero variance. Maps were generated in QGIS version 3.18.3.

**Fig. 2.**
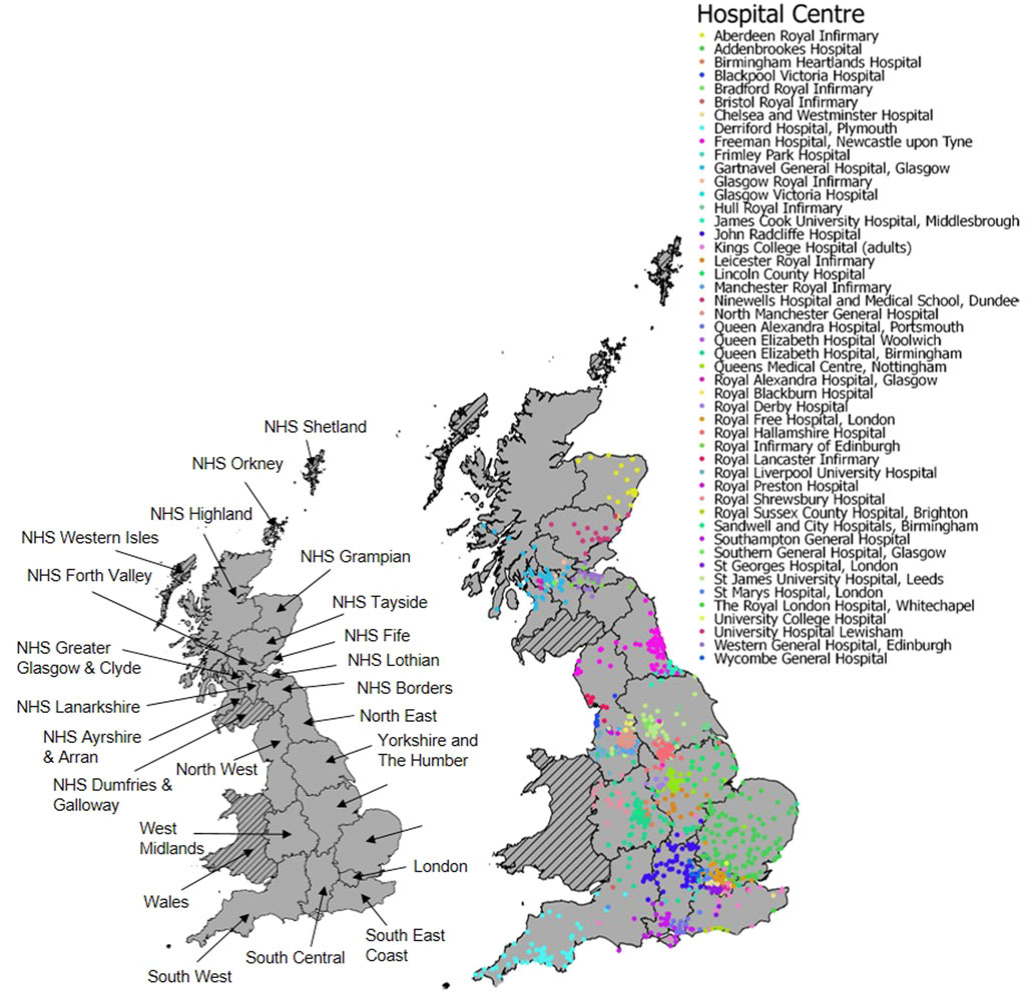
Location of 3829 HCV-positive study participants recruited in Great Britain by HCVRUK between March 2012 and September 2014. Residential postcodes coloured according to the recruiting hospital centre. Insert shows the location of each regional health authority area. Patients residing in Wales, NHS Shetland and NHS Western Isles were excluded due to small numbers. No patients were recruited from NHS Dumfries & Galloway or NHS Orkney.

Mixed-effects univariable logistic regression was used to identify factors associated with severe liver disease: demographic (age, sex, ethnicity, country of birth), social/behavioural (probable route of HCV infection, history of heavy alcohol consumption) and clinical (BMI, diabetes, HCV-genotype and HIV-coinfection). All variables, including age, were treated as categorical in order to understand their specific effects. A variable differentiating patient residence in England or Scotland was included to adjust for country-level differences in demography and healthcare provision. Hospital centres and LHAs were fitted as random effects to account for patient clustering using a crossed design. Patient hospital centres were not consistently nested within health authority areas of residence, with some patients enrolled at hospitals out with their LHA area (Fig. 2). Factors were fitted as binary or categorical (see Supplementary Tables S1–S3 for details).

Mixed-effects multivariable logistic regression was performed to investigate (i) the independent association of patient risk factors with severe liver disease and (ii) how much of the geographical variation in severe liver disease was attributable to patient factors. Statistical interactions were investigated post-hoc among significant factors in fixed-effect multivariable logistic regression models. A Bonferroni correction was applied to an initial significance threshold of *P* < 0.05 yielding a corrected threshold of *P* < 0.0026. An approximate Variation Partition Coefficient (VPC) was estimated to gauge how much of the total variance in the odds of severe liver disease was attributable to differences between hospital centres and LHAs (assuming individual-level residual variance = *π*^2^/3).

Statistical modelling was performed in R using ‘glmer’ (lme4 package[18]) and ‘glm’ for mixed-effect and fixed-effect only models respectively. Analyses were based on a subset of 2851 patients who had complete information across all variables. Patient distributions were unchanged following these exclusions (see Supplementary Material Tables S1–S3).

## Results

### Patient distributions: demographics, severe liver disease and patient risk factors

Figure 2 shows the distribution of study patients across England and Scotland according to residential postcode and enrolling hospital centre. Most of the 3829 patients (75%; *n* = 2858) were resident in England. The East Midlands of England represented the largest LHA, recruiting 12% (*n* = 477) of study patients. Most patients (64%; *n* = 2444) had mild liver disease at enrolment, with the remaining patients (36%; *n* = 1385) classified as having severe liver disease, and most patients were untreated (71%, *n* = 2730). Of the treated patients (29%; 1099), 26% were previously treated with a non-DAA, and 1.9% had been treated with a DAA at the time of enrolment following early access to DAA therapy as part of the Scottish or English Early Access Programmes or clinical trials. For a small number of patients (a further 0.24%) the treatment drug was unknown.

The median patient age was 49 years (IQR: 56 y–41 y = 15 y) and 70% (*n* = 2696) of patients were male. Most patients (77%; 2935) were of white ethnicity, born in the UK (75%; 2884), most likely infected via IDU (62%; 2358), and had no self-reported previous history of heavy alcohol consumption (57%; 2192). The most prevalent BMI group was ‘normal’ (36%; *n* = 1367), although high BMI was also relatively prevalent (31%; *n* = 1195). Most patients were non-diabetic (89%; *n* = 3415).

HCV-genotype 1 was the most prevalent HCV subtype (52%, *n* = 1978), whilst HCV GT-3 was identified in 34% (*n* = 1300) of patients. Of the remaining patients with genotyped infection, 4% (*n* = 167) were infected with HCV GT-2 and 3% (*n* = 123) with HCV GT-4. A small number of HCV GT-5, HCV GT-6, and mixed infections were also observed. The infecting HCV virus was not genotyped in 230 (6%) of patients. Most patients were HIV-negative (90%; *n* = 3428). See Supplementary Tables S1–S3.

### Geographical distribution of severe liver disease and treatment coverage

**Fig. 3.**
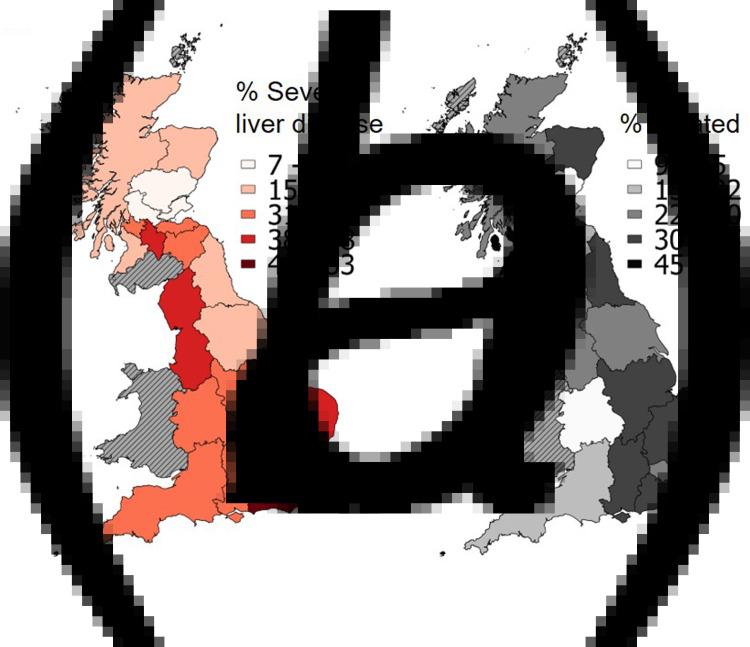
Regional variation in (a) the prevalence of cirrhosis and (b) the prevalence of untreated individuals. Among 3829 hepatitis C positive patients recruited by HCVRUK from across England and Scotland. See also Supplementary Material Table S4.

Geographical variation in the proportion of the study patient population with severe liver disease across LHA was moderate (CV 36%, 95% CI 27%–52%). The highest proportion of severe liver disease of 53% was observed in the South East Coast region, England, compared to a low of 7% in NHS Fife, Scotland (Fig. 3a). Treatment history was also highly variable across LHA areas (CV 40, 95% CI 31–58) and broadly mirrored disease severity (Fig. 3b). In most areas (except for NHS Forth Valley and NHS Ayrshire and Arran, Scotland), more than 50% of patients were untreated. Areas with high proportions of severe liver disease, for example, the South East Coast (53%), North West (48%) and East (46%) of England had moderate levels of treatment coverage (37%, 30% and 37%, respectively). In contrast, areas with low proportions of severe liver disease had the lowest levels of treatment, for example NHS Tayside in Scotland. Exceptions were noted; for example, NHS Forth Valley had a low proportion with severe liver disease (14%) but high treatment coverage (52%). See Supplementary Table S4.

Assessing the prevalence of severe liver disease among all patients attending HCV specialist services in Scotland during the study period (based on the Scottish HCV Clinical database) showed a similar ranking by location. Clear anomalies were observed for NHS Borders and NHS Greater Glasgow and Clyde (Supplementary Table S5).

### Geographical distribution of putative risk factors

Geographical variation in putative risk factors among the study patient population across LHA varied from a low CV of 9.3% (95% CI 7%–14%) for age group, to a high CV of 146% (95% CI 113%–216%) for HIV infection status.

**Fig. 4.**
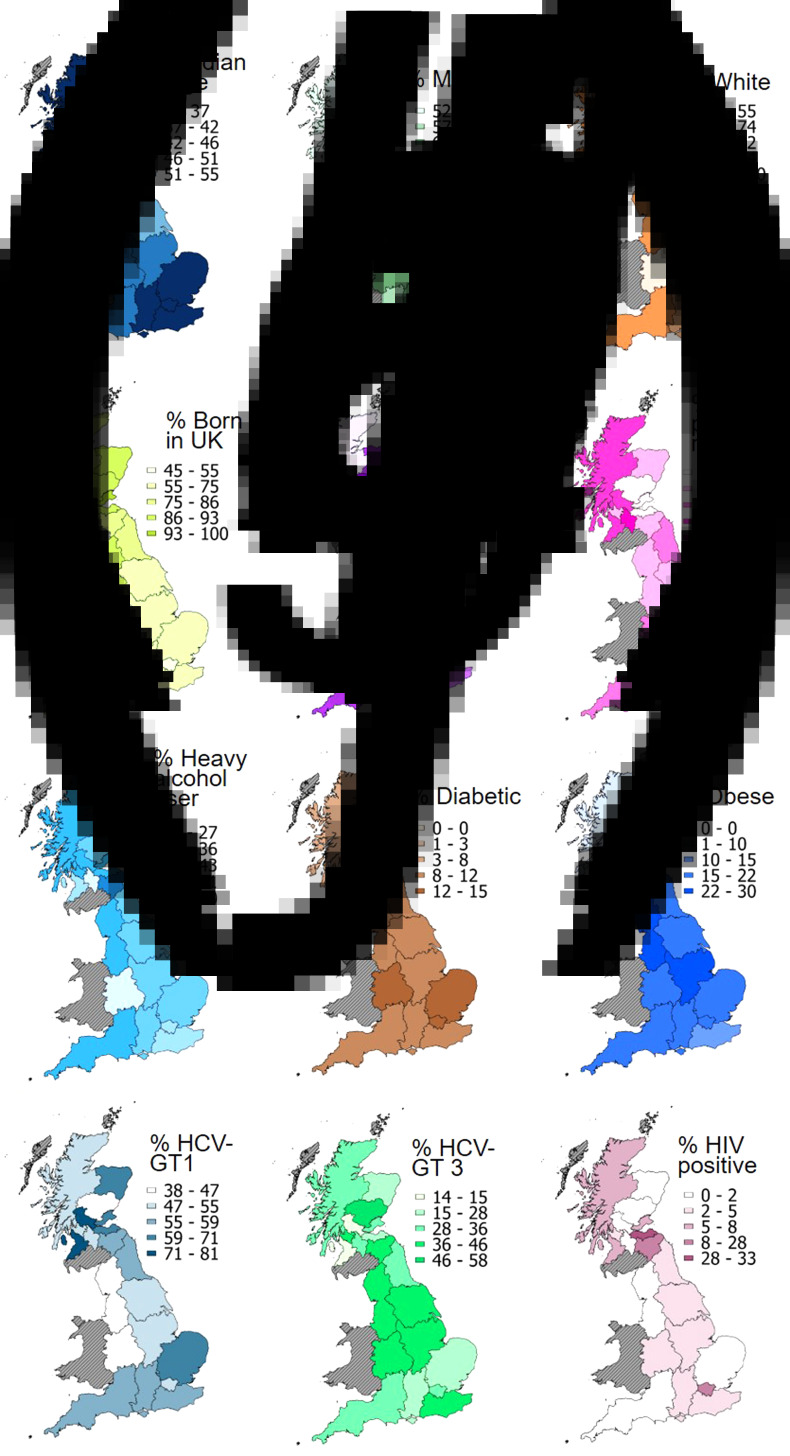
Regional variation in potential demographic, social and health-related correlates of severe liver disease. Among 3829 hepatitis C positive patients recruited by HCVRUK from across England and Scotland. See also Supplementary Material Tables S6–S7.

Patient age ranged from a median 37 years (IQR = 16 years) in NHS Tayside, Scotland, to 55 years in South East Coast, England (IQR = 13.5) and NHS Lanarkshire, Scotland (IQR = 12.5) (Fig. 4a). Patients were predominantly male in all areas, ranging from 86% in NHS Fife, Scotland, to 52% in NHS Forth Valley, Scotland (Fig. 4b). Although white ethnicity dominated, representing 100% of recruited patients in NHS Ayrshire and Arran, NHS Fife, and NHS Forth Valley in Scotland, other ethnic groups were prominent in some areas of England, with 54% of patients in London and 45% in West Midlands being non-white (Fig. 4c). The distribution of place of birth closely mirrored ethnicity (Fig. 4d).

The proportions of study patients with IDU as the most probable route of HCV infection was overall greater in Scotland than England (75% *vs.* 58%), with the highest levels in NHS Tayside (88%) and NHS Borders (85%) (Fig. 4e). However, although IDU was the most prevalent route in all areas of England, the blood products route had a higher prevalence in several areas of Scotland: NHS Ayrshire and Arran (42%), NHS Highland (38%) and NHS Lanarkshire (55%) (Fig. 4f). A high prevalence of individuals with unknown route of infection was observed for London and the West Midlands, England (24% and 33% respectively), where there was also high representation of patients born outside of the UK (Fig. 4d).

Fifty per cent or more of patients had a history of heavy alcohol consumption in three areas of Scotland (Fig. 4g): NHS Borders (60%), NHS Fife (50%) and NHS Grampian (51%). The highest prevalence of diabetes was observed in West Midlands, England (15%) (Fig. 4h) whilst the highest prevalence of obesity was observed for NHS Lanarkshire, Scotland (30%) (Fig. 4i). Diabetes and obesity both showed a marked degree of geographical variation when compared to other patient risk factors (CV 48, 95% CI 37–70 and CV 44, 95% CI 34–65 respectively).

Geographically, HCV GT-1 was the most prevalent genotype in all areas (except for NHS Tayside, Scotland), with the highest levels found in several regional health boards of Scotland (Fig. 4j): NHS Forth Valley (81%), NHS Ayrshire & Arran (77%) and NHS Fife (71%). In NHS Tayside, Scotland, HCV GT-3 was more prevalent than HCV GT-1 (58% *vs.* 38%, respectively) (Fig. 4k). HCV GT-1 and GT-3 were equally prevalent in the North West (47% *vs.* 46%, respectively) and West Midlands (46% *vs.* 44%, respectively), England. A marked geographical variation in HIV coinfection was found, when compared to other patient risk factors (CQV = 56, 95% CI 28–85). Most areas had a HIV-coinfection prevalence less than 9%; however, a relatively high HIV-coinfection prevalence was observed in NHS Lothian (33%) and NHS Borders (28%), Scotland and London, England (11%) (Fig. 4l). See Supplementary Tables S6-S7.

### Investigating patient risk factors and geographical variation in severe liver disease

Univariable mixed-effects modelling revealed several factors significantly associated with the odds of severe liver disease: age, sex, ethnicity, probable route of HCV infection, history of heavy alcohol consumption, BMI, diabetes and HCV-genotype (see Table 1).

**Table 1.** Factors associated with severe liver disease; mixed-effects logistic regression model results

Factor	Level	Number (%) without severe disease	Number (%) with severe disease	Unadjusted^a^ OR (95% CI; *P* value)	Adjusted^b^ OR (95% CI; *P* value)
Age (years)	<46	871 (48.15)	205 (19.67)	Reference	Reference
	46–59	742 (41.02)	578 (55.47)	2.88 (2.44–3.41; *P* < 0.001)*	3.06 (2.48–3.76; *P* < 0.001)*
	≥60	196 (10.83)	259 (24.86)	4.75 (3.82–5.91; *P* < 0.001)*	5.64 (4.26–7.48; *P* < 0.001)*
Sex	Female	574 (31.73)	247 (23.70)	Reference	Reference
	Male	1235 (68.27)	795 (76.30)	1.45 (1.24–1.69; *P* < 0.001)*	1.58 (1.29–1.94; *P* < 0.001)*
Ethnicity	White	1396 (77.17)	810 (77.74)	Reference	Reference
	Asian	106 (5.86)	92 (8.83)	1.29 (0.98–1.71; *P* = 0.071)	1.13 (0.64–1.99; *P* = 0.684)
	Black	39 (2.16)	10 (0.96)	**0.49 (0.27–0.89; *P* = 0.019)**	**0.31 (0.13–0.73; *P* = 0.008)**
	Other^c^	268 (14.81)	130 (12.48)	0.68 (0.55–0.84; *P* < 0.001)*	1.14 (0.71–1.83; *P* = 0.588)
Country of birth	Non-UK	417 (23.05)	247 (23.70)	Reference	Reference
	UK	1392 (76.95)	795 (76.30)	**1.25 (1.05–1.49; *P* = 0.014)**	1.1 (0.71–1.71; *P* = 0.676)
Probable route of infection	Injecting drug use	1199 (66.28)	600 (57.58)	Reference	Reference
	Blood/blood products	209 (11.55)	154 (14.78)	**1.36 (1.1–1.67; *P* = 0.005)**	**1.40 (1.06–1.87; *P* = 0.019)**
	Other^d^	157 (8.68)	113 (10.84)	1.13 (0.88–1.44; *P* = 0.339)	1.27 (0.93–1.73; *P* = 0.137)
	Unknown	244 (13.49)	175 (16.79)	**1.29 (1.06–1.58; *P* = 0.013)**	1.36 (1–1.85; *P* = 0.053)^†^
Heavy alcohol consumption^e^	No	1133 (62.63)	524 (50.29)	Reference	Reference
	Yes	676 (37.37)	518 (49.71)	1.88 (1.63–2.17; *P* < 0.001)*	1.94 (1.60–2.35; *P* < 0.001)*
Body Mass Index (BMI)	Normal	610 (46.38)	413 (29.37)	Reference	Reference
	Low	55 (3.04)	18 (1.73)	1.01 (0.6–1.7; *P* = 0.965)	0.87 (0.48–1.59; *P* = 0.652)
	High	839 (33.72)	306 (39.64)	1.79 (1.5–2.13; *P* < 0.001)*	1.73 (1.41–2.12; *P* < 0.001)*
	Obese	305 (16.83)	305 (29.27)	2.61 (2.14–3.18; *P* < 0.001)*	2.81 (2.21–3.56; *P* < 0.001)*
Diabetes	No	1712 (94.64)	843 (80.90)	Reference	Reference
	Yes	97 (5.36)	199 (19.10)	3.51 (2.78–4.42; *P* < 0.001)*	2.75 (2.04–3.69; *P* < 0.001)*
HCV-genotype^f^	GT-1	1039 (57.44)	537 (51.54)	Reference	Reference
	GT-3	602 (33.28)	431 (41.36)	1.59 (1.36–1.86; *P* < 0.001)*	1.75 (1.43–2.14; *P* < 0.001)*
	Other	168 (9.29)	74 (7.10)	0.89 (0.68–1.16; *P* = 0.387)	0.71 (0.5–0.99; *P* = 0.042)^†^
HIV coinfection	No	1719 (95.02)	995 (95.49)	Reference	Reference
	Yes	90 (4.98)	47 (4.51)	0.81 (0.57–1.16; *P* = 0.258)	1.31 (0.84–2.03; *P* = 0.233)
Country of residence	England	1291 (71.37)	829 (79.56)	Reference	Reference
	Scotland	518 (28.63)	213 (20.44)	0.62 (0.28–1.35; *P* = 0.228)	0.67 (0.33–1.38; *P* = 0.28)

Results highlighted in bold font were considered statistically significant at the 5% level.

a Unadjusted odds ratios (OR): mixed-effects logistic regression adjusting for a single patient risk factor and including hospital centre and regional health authority random effects.

b Adjusted OR: multivariable mixed-effects logistic regression adjusting for all patient risk factors and including hospital centre and regional health authority random effects.

c Mixed/multiple ethnic groups.

d Comprised predominantly of individuals with no known risk factor and a small number of other routes such as a known hepatitis C positive sexual partner, dental, tattoo/piercing, intranasal exposure, having lived abroad or perinatal exposure.

e Self-reported history of heavy alcohol consumption.

f Other HCV genotypes: predominantly GT-2 and GT-4 and a small number of GT-5, GT-6 and mixed genotype infections.

* *P* value significant applying Bonferroni corrected alpha-level of 0.0026.

† 95% CI borders or overlaps 1.0 and so the result is considered insignificant.

Adjusting for all covariates in multivariable mixed-effects models, several independent risk factors were identified: age 46–59 y (OR_adj_ 3.06) and ≥60 y (OR_adj_ 5.64) relative to <46 y, male relative to female sex (OR_adj_ 1.58), probable route of infection via blood products relative to IDU route (OR_adj_ 1.40), history of heavy alcohol consumption relative to no history (OR_adj_ 1.94), high BMI (OR_adj_ 1.73) or obese (OR_adj_ 2.81) relative to normal BMI, diabetic relative to not diabetic (OR_adj_ 2.75) and infection with HCV GT-3 relative to HCV GT-1 (OR_adj_ 1.75). Patients of black ethnicity were less likely to have severe liver disease (OR_adj_ 0.31) relative to patients of white ethnicity. Statistical interactions were examined between (i) BMI and diabetes, (ii) ethnicity and HCV-genotype, (iii) ethnicity and probable route of infection and (iv) country of birth and route of infection, and did not reveal evidence of effect modification at a 5% level of significance. Treatment history at enrolment was significantly associated with severe disease (OR = 2.42, 95% CI = 2.07–2.82, p < 0.001) but was not included as a risk factor, since disease severity is expected to determine treatment status.

Country of residence (Scotland *vs.* England) was not associated with disease severity (OR_adj_ 0.67). Despite a lack of association at the country-level, significant geographical variation in the odds of recruited patients having severe liver disease was found across LHAs and hospital centres (LRT *P* < 0.001). The random effect variances were 41.19% for hospital centres and 16.11% for LHAs. An approximate estimate of the Variant Partition Coefficients suggested most of the variation in the odds of a patient having severe disease was attributed to patient risk factors, with 10.66% and 4.17% of the remaining variation attributed to unexplained variation between hospital centres and LHAs respectively. See Table 1 for full details of the statistical modelling results.

## Discussion

Our study exploits the broad geographical coverage and rich patient metadata collected by HCVRUK to provide insight into the distribution of HCV-associated severe liver disease (as measured by cirrhosis) and associated risk factors, among patients attending specialist hospital clinics in the pre-DAA era across England and Scotland, UK. Data collated through HCVRUK were not intended to provide a random sample of the HCV-infected population or those accessing liver centres. However, these data provide the only means currently to (i) assess the geographical distribution of HCV-associated cirrhosis among patients attending liver centres in the pre-DAA era and (ii) quantify associated risk factors. These data provide baseline metrics to support the ongoing monitoring of changes in HCV-related severe disease burden, and have implications for improved understanding of disease pathogenesis and the design of targeted preventative measures.

We identified significant geographical variation in the proportion of patients enrolled into HCVRUK with severe liver disease. These findings are consistent with estimates of variation in both risk factors for liver disease and healthcare provision in England, including hepatitis C-related hospital admissions [1]. The highest proportion of severe liver disease was observed for South East Coast, England, with the lowest in NHS Fife, Scotland. Overall, a lower proportion of severe liver disease was observed for Scotland, in line with high testing rates and an increased uptake of HCV therapy in recent years among PWID (see The Needle Exchange Surveillance Initiative [19]). Reductions in the underlying prevalence of chronic HCV infections in Scotland were observed in the years prior to COVID-19 [19], likely reflecting the hepatitis C Action Plan's initiative to specifically target prevention at the PWID risk group [20].

The geographical coverage of previous treatment in this study generally mirrored the proportion of severe disease, reflecting a period during which newline DAA therapies were not in mainstream use [21]. One notable exception was NHS Forth Valley in Scotland, which ranked second highest for proportions of recruited patients who had previously received treatment, whilst ranking second lowest for proportions with severe disease. Also of note was NHS Tayside in Scotland, which ranked the lowest for the proportion treated whilst also ranking low for the proportion with severe disease, most likely reflecting high levels of HCV screening among PWID (as indicated by the high proportion of patients with IDU as the probable route of infection) and therefore capturing asymptomatic infections. It remains to be seen how recent progress in reducing overall liver disease burden in the UK, and the long-term impact of DAA therapy deployment, will be offset by indirect impacts of the COVID-19 epidemic such as through a diminished access to harm reduction programmes [22].

Our study identified several independent risk factors for severe liver disease. We found an increased risk of severe liver disease with older age, an unsurprising finding given that older age at the point of infection is known to enhance speed of disease progression [11, 12, 23], and older individuals may have been infected for a longer duration. Male biological sex was also an independent risk factor, in line with previous studies [12]. Our study revealed a lower risk of severe disease associated with black ethnicity relative to white ethnicity, although we note the small numbers of patients representing this group and the potential for selection bias if people of black ethnicity with severe disease are less likely to attend liver centres than people of white ethnicity. Our finding supports a previous study from the USA [24], but contrasts with previous reports from the UK of higher rates of primary and severe liver disease among ethnic minorities, perhaps reflecting differentials in healthcare access and/or infection prevalence [25, 26]. The reason for our finding is unclear and warrants further investigation.

Several social-related factors were independently associated with severe liver disease. The acquisition of HCV infection through blood products was associated with a greater risk of severe disease relative to IDU, as previously reported for a large study investigating the impact of infection route on cirrhosis risk in France [27]. Haemophiliac patients infected with HCV in the 1980s had received regular treatment with factor VIII, each dose of which had been obtained from several donors. It is likely that infection through such blood products resulted in a higher exposure inoculum, multiple exposures to different strains, as well as being associated with a longer duration of infection, thus leading to severe liver disease [28]. Patients with an unknown route of HCV infection were more likely to have severe disease than those infected through IDU, as reported by others, perhaps reflecting a lack of awareness of their risk of disease and delayed diagnosis [27]. Genotype prevalence is known to vary by infection route, with a higher prevalence of HCV GT-1b among blood transfusion recipients and those with unknown route [29]. Our analyses suggest the effect of blood product route on disease severity was independent of HCV genotype. Our study also supports the well-recognised role of heavy alcohol consumption in enhancing the risk of HCV-associated liver cirrhosis [11, 12, 15, 27, 30].

Viral and comorbid risk factors were also identified. With respect to HCV-genotype, its role in disease severity has been explored in many studies with conflicting results. Any associations with disease severity may be confounded by the duration and route of infection and the likelihood of response to treatment. Several early studies did not find genotype to be associated with disease severity [11, 12, 15, 31], whilst others found an association of HCV GT-1, particularly HCV GT-1b, with advanced fibrosis and liver-associated death [14, 32]. Our finding of a greater risk of severity for HCV GT-3 relative to HCV GT-1 is in line with recent studies reporting a more rapid progression to cirrhosis and mortality associated with HCV GT-3 compared to HCV GT-1 [24, 33, 34].

HIV-coinfection is associated with greater rates of fibrosis progression in HCV patients, especially in those with poorly controlled infection [11, 30, 35]. The role of HIV was not highlighted by our study, likely owing to low statistical power but may also have been affected by improved treatment of HIV. Findings for other comorbidities were as expected; elevated BMI increased the risk of severe liver disease, likely as a result of co-existing non-alcoholic fatty liver disease. Both obesity and diabetes were independently associated with severe liver disease.

Our study revealed no significant difference in the risk of severe liver disease between patients enrolled in England or Scotland, although regional differences were observed at a finer-scale. The geographical pattern of risk factors suggests older age, HCV GT-3, male sex and blood product route likely contributed to the relatively high proportion of severe liver disease among clinic attendees in South East Coast England. However, the apparent lack of a Scottish effect is at odds with the highest prevalences of heavy alcohol consumption and IDU route observed among the Scottish study patients. The ‘Scottish effect’ is a phenomenon related to socio-economic and lifestyle factors hypothesised to contribute to unexplained excess mortality [36], and thus is anticipated to be reflected in the burden of diseases associated with health inequalities such as hepatitis C. Of note is the low prevalence of obesity which is recognised to be higher in Scotland than other UK nations [37]; the study population is however expected to over-represent individuals exhibiting poor lifestyle choices, and for example drug-induced suppression of appetite. Furthermore, a relatively greater risk of severe liver disease was associated with the blood product route rather than IDU, and in regions of Scotland with high IDU route, the risk may have been offset by higher prevalences of HCV GT-1 than HCV GT-3. It was not possible to measure the extent of any potential biases in risk factor distributions introduced by the study recruitment process.

Most of the geographical variation in the proportion of severe liver disease was explained by patient risk factors, however, 10.66% and 4.17% of the unexplained variation was attributable to hospital centres and LHA areas respectively. Two of the seven adult liver transplant units in the UK represented a greater than average risk, suggesting some of the between-centre variation may be an artefact of centre type. It should be noted that the study is not expected to accurately reflect prevalence of severe liver disease among the underlying HCV-infected population, which may be biased by regional differences in HCV diagnostic testing practices, and by healthcare usage and provision, with the predominantly hospital-based recruitment expected to under-represent some risk groups such as PWID. Regional differences in the proportion of severe liver disease may therefore signify differences in how well-equipped healthcare services are, the timeliness of HCV diagnosis and healthcare access. Historically, provision of liver disease services was recognised to vary geographically, with premature mortality from liver disease varying 7.7-fold across local NHS bodies during 2013–15, seemingly for reasons other than social deprivation [1]. Such geographical variation extended to HCV specialist services, where it was associated with inequalities in treatment access [38]. It was not possible in our study to formally evaluate differences in recruitment practice and success rates across centres. We note however that rankings of proportions with severe disease by regional location closely mirrored the prevalence among all patients attending liver services, albeit with two anomaly regions (as assessed for Scotland).

Our study has several other important limitations. Firstly, the cross-sectional design does not consider person-time at risk. Ascribing causation is also limited by unknown sequential timings of exposures and outcome. Furthermore, reinfections of HCV are common, and the temporal association of infections with heterotypic genotypes, the development of severe disease, and the point of enrolment into the study, are not known. Secondly, some of the data accrued through patient interviews may lead to some occurrences of imprecise or subjective information, such as those relating to alcohol consumption and the most likely route of infection. Thirdly, some caution is warranted when interpreting results based on small patient numbers. Fourthly, our study does not account for changes in the residential location of patients over the course of infection and disease stages. Finally, HCV-negative patients were not captured; these patients may have been treated, cured and discharged from hospital during the period of patient enrolment. Therefore, high relative occurrences of severe liver disease may coincide with high levels of HCV diagnostic testing and/or treatment success. On the other hand, we note that our study also does not account for survival bias, which may lead to under-estimated occurrence of severe disease.

Improved understanding of geographical variations in healthcare provision may enable optimal allocation of public health resources, to ensure continued uptake and coverage of new HCV antiviral therapies. Analysis at a more geographically granular level, together with socioeconomic factors, would be optimal for informing interventions at a more refined local community scale.

## Conclusions

Our study found significant geographical variation in the proportion of severe liver disease and associated risk factors among HCV-infected liver service attendees recruited into the HCVRUK study population from 48 centres across the UK. Our study provides further support for older age, male gender, heavy alcohol consumption, and high-to-obese BMI as predictors of hepatitis C-related severe liver disease, and unexpectedly revealed HCV GT-3 and blood product route as independent risk factors and a relatively lower risk for black ethnicity. Whilst geographical variation in risk factor prevalence explained a large proportion of the variation in severe liver disease, 10.66% was attributable to unexplained differences between hospital centres. Improved understanding into unexplained geographical variation in severe liver disease across the UK is needed, and whether this is attributed to HCV-related healthcare usage and provision, in order for patient services to appropriately accommodate locally defined risk groups.

## Data Availability

The patient level data used in this study are considered personally identifiable and confidential information and therefore cannot be made publically available in line with the General Data Protection Regulation (GDPR). The data are however available to researchers upon successful application to the HCV Research UK Tissue and Data Access Committee (www.hcvresearchuk.org). Further information on HCV Research UK cohort data can be found here: https://www.ncbi.nlm.nih.gov/pmc/articles/PMC5837619/.

## Supplementary material

For supplementary material accompanying this paper visit http://doi.org/10.1017/S0950268823000377. click here to view supplementary material

## References

[1] Public Health England (2017) 2nd Atlas of variation in risk factors and healthcare for liver disease in England. Available at https://fingertips.phe.org.uk/profile/atlas-of-variation [Last accessed 15/01/2023].

[2] Public Health England (2014) Hepatitis C in the UK 2014 report. Available at http://www.hcvaction.org.uk/resource/hepatitis-c-uk-2014-report [Last accessed 15/01/2023].

[3] Harris HE (2022) Hepatitis C in England, 2022: Working to eliminate hepatitis C as a public health problem. Full report. March, 2022. UK Health Security Agency, London. Available at https://assets.publishing.service.gov.uk/government/uploads/system/uploads/attachment_data/file/1057271/HCV-in-England-2022-full-report.pdf [Last accessed 15/01/2023].

[4] McLeod (2019) Surveillance report. Surveillance of hepatitis C testing, diagnosis and treatment in Scotland, 2019 update. Available at https://hpspubsrepo.blob.core.windows.net/hps-website/nss/2834/documents/1_hcv-testing-diagnosis-treatment-scotland-2018.pdf [Last accessed 15/01/23].

[5] Public Health Scotland (2022) Surveillance of hepatitis C in Scotland. Progress on elimination of hepatitis C as a major public health concern: 2022 update. Available at https://www.publichealthscotland.scot/media/15549/surveillance-of-hepatitis-c-in-scotland-oct22.pdf [Last accessed 15/01/2023].

[6] WHO (2018) Progress Report on Access to Hepatitis C Treatment. Focus on overcoming barriers in low-and middle-income countries. Available at https://apps.who.int/iris/bitstream/handle/10665/260445/WHO-CDS-HIV-18.4-eng.pdf [Last accessed 15/01/23].

[7] HCVAction (2020) HCV action webinar: hepatitis C services in Scotland during and beyond the COVID-19 outbreak August 2020. Summary Report. Available at http://www.hcvaction.org.uk/sites/default/files/resources/HCV%20Action%20Scotland%20Webinar%20Summary%20Report.pdf [Last accessed 15/01/23].

[8] DHSC and PHE (2021) Guidance. COVID-19: guidance for commissioners and providers of services for people who use drugs or alcohol. Available at https://www.gov.uk/government/publications/covid-19-guidance-for-commissioners-and-providers-of-services-for-people-who-use-drugs-or-alcohol/covid-19-guidance-for-commissioners-and-providers-of-services-for-people-who-use-drugs-or-alcohol [Last accessed 15/01/23].

[9] Harris HE (2020) Hepatitis C in England, 2020 report: Working to eliminate hepatitis C as a major public health threat, May, 2020. Public Health England, London. Available at http://www.hcvaction.org.uk/sites/default/files/resources/HCV_in_England_2020_Report_270520.pdf [Last accessed 15/01/23].

[10] WHO (2016) Global Health Sector Strategy on Viral Hepatitis 2016–2021. Towards Ending Viral Hepatitis. Available at https://www.who.int/publications/i/item/WHO-HIV-2016.06 [Last accessed 15/01/23].

[11] Pol S (1998) Predictive factors for development of cirrhosis in parenterally acquired chronic hepatitis C: a comparison between immunocompetent and immunocompromised patients. Journal of Hepatology 29, 12–19. 9696487 10.1016/s0168-8278(98)80173-6

[12] Poynard T, Bedossa P and Opolon P (1997) Natural history of liver fibrosis progression in patients with chronic hepatitis C. The Lancet 349, 825–832. 10.1016/s0140-6736(96)07642-8 9121257

[13] Graham CS (2001) Influence of human immunodeficiency virus infection on the course of hepatitis C virus infection: a meta-analysis. Clinical Infectious Diseases 33, 562–569. 11462196 10.1086/321909

[14] Garcia-Samaniego J (1997) Influence of hepatitis C virus genotypes and HIV infection on histological severity of chronic hepatitis C. The hepatitis/HIV Spanish Study Group. American Journal of Gastroenterology 92, 1130–1134. 9219784

[15] Serfaty L (1997) Risk factors for cirrhosis in patients with chronic hepatitis C virus infection: results of a case-control study. Hepatology 26, 776–779. 9303512 10.1002/hep.510260334

[16] McLauchlan J (2017) Cohort profile: the hepatitis C Virus (HCV) research UK clinical database and biobank. International Journal of Epidemiology 46, 1391–1391h. 28338838 10.1093/ije/dyw362 PMC5837619

[17] ONS (2018) ONS Postcode Directory (November 2018). Available at: https://geoportal.statistics.gov.uk/datasets/ons::ons-postcode-directory-november-2018-1/about [Last accessed 25/03/23].

[18] Bates D (2015) Fitting linear mixed-effects models using lme4. Journal of Statistical Software 67, 1–48.

[19] Health Protection Scotland (2019) The Needle Exchange Surveillance Initiative (NESI): Prevalence of Blood-Borne Viruses and Injecting Risk Behaviours among People Who Inject Drugs (PWID) Attending Injecting Equipment Provision (IEP) Services in Scotland, 2008–09 to 2017–18. Glasgow: Health Protection Scotland. Available at https://www.hps.scot.nhs.uk/web-resources-container/needle-exchange-surveillance-initiative-nesi-2008-09-to-2017-18 [Last accessed 15/01/23].

[20] Health Protection Scotland (2019) Scotland's Hepatitis C Action Plan: achievements of the first decade and proposals for a Scottish Government strategy (2019) for the elimination of both infection and disease. Taking advantage of outstanding new therapies. Available at http://www.hcvaction.org.uk/sites/default/files/resources/Scotland%20Proposals%20for%20a%20Scottish%20Government%20Strategy.pdf [Accessed 15/01/23].

[21] Public Health England (2014) Improving access to hepatitis C treatment: turning evidence into practice. Available at https://www.gov.uk/government/publications/treating-substance-misuse-and-related-harm-turning-evidence-into-practice/improving-access-to-hepatitis-c-treatment-turning-evidence-into-practice [Last accessed 15/01/23].

[22] Scottish Drugs Forum (2020) Guidance on Contingency Planning for People who use Drugs and COVID-19. Available at https://www.sdf.org.uk/wp-content/uploads/2020/05/Guidance-on-Contingency-Planning-for-PWUD-and-COVID19-V2.0-May-2020.pdf [Last accessed 15/01/23].

[23] Di Bisceglie AM (1991) Long-term clinical and histopathological follow-up of chronic posttransfusion hepatitis. Hepatology 14, 969–974. 1959884 10.1016/0270-9139(91)90113-a

[24] McCombs J (2014) The risk of long-term morbidity and mortality in patients with chronic hepatitis C: results from an analysis of data from a department of veterans affairs clinical registry. JAMA Internal Medicine 174, 204–212. 24193887 10.1001/jamainternmed.2013.12505

[25] Mann AG (2008) Hepatitis C in ethnic minority populations in England. Journal of Viral Hepatitis 15, 421–426. 18208498 10.1111/j.1365-2893.2007.00958.x

[26] Ladep NG (2014) Incidence and mortality of primary liver cancer in England and Wales: changing patterns and ethnic variations. World Journal of Gastroenterology 20, 1544–1553. 24587630 10.3748/wjg.v20.i6.1544 PMC3925863

[27] Roudot-Thoraval F (1997) Epidemiological factors affecting the severity of hepatitis C virus-related liver disease: a French survey of 6,664 patients. The study group for the prevalence and the epidemiology of hepatitis C virus. Hepatology 26, 485–490. 9252163 10.1002/hep.510260233

[28] Di Bisceglie M (1998) Hepatitis C. The Lancet 351, 351–355. 10.1016/S0140-6736(97)07361-3 9652633

[29] Pawlotsky JM (1995) Relationship between hepatitis C virus genotypes and sources of infection in patients with chronic hepatitis C. Journal of Infectious Diseases 171, 1607–1610. 7769300 10.1093/infdis/171.6.1607

[30] Benhamou Y (1999) Liver fibrosis progression in human immunodeficiency virus and hepatitis C virus coinfected patients. Hepatology 30, 1054–1058. 10498659 10.1002/hep.510300409

[31] Puoti M (2001) Liver fibrosis progression is related to CD4 cell depletion in patients coinfected with hepatitis C virus and human immunodeficiency virus. Journal of Infectious Diseases 183, 134–137. 11087200 10.1086/317644

[32] Yee TT (2000) The natural history of HCV in a cohort of haemophilic patients infected between 1961 and 1985. Gut 47, 845–851. 11076885 10.1136/gut.47.6.845 PMC1728144

[33] Wu N (2020) Impact of hepatitis C virus genotype 3 on liver disease progression in a Chinese national cohort. Chinese Medical Journal 133, 253–261. 31934936 10.1097/CM9.0000000000000629 PMC7004615

[34] Bochud PY (2009) Genotype 3 is associated with accelerated fibrosis progression in chronic hepatitis C. Journal of Hepatology 51, 655–666. 19665246 10.1016/j.jhep.2009.05.016

[35] Mohsen AH (2002) Hepatitis C and HIV-1 coinfection. Gut 51, 601–608. 12235089 10.1136/gut.51.4.601 PMC1773386

[36] Cowley J, Kiely J and Collins D (2016) Unravelling the Glasgow effect: the relationship between accumulative bio-psychosocial stress, stress reactivity and Scotland's health problems. Preventive Medicine Reports 4, 370–375. 27512652 10.1016/j.pmedr.2016.08.004 PMC4979043

[37] The Scottish Governement (2010) The Scottish Health Survey. Available at https://www.gov.scot/binaries/content/documents/govscot/publications/research-and-analysis/2010/08/scottish-health-survey-topic-report-uk-comparisons/documents/0103950-pdf/0103950-pdf/govscot%3Adocument/0103950.pdf [Last accessed 15/01/23].

[38] APPHG (2010) In The Dark: An audit of hospital hepatitis C services across England. Available at http://www.appghep.org.uk/reports [Last accessed 15/01/23].

