# Supplementary material for: Geographical variation in hepatitis C-related severe liver disease and patient risk factors: a multicentre cross-sectional study
Source: Epidemiol Infect. 2023 Mar 14;151:e59. doi: 10.1017/S0950268823000377 (PMC10126891; doi:10.1017/S0950268823000377)
Supplement: Supplementary file 1 [file S0950268823000377sup001.docx]

**Geographical variation in Hepatitis C-related severe liver disease and patient risk factors: A multicentre cross-sectional study**

**Supplementary material**

Sema Nickbakhsh^1,a,b*^, E. Carol McWilliam Leitch^1,c^, Shanley Smith^2^, Chris Davis^1^, Sharon Hutchinson^2^, William L. Irving^3^, John McLauchlan^1^, Emma C. Thomson^1*^

Affiliations:

^1^MRC-University of Glasgow Centre for Virus Research, College of Medical, Veterinary and Life Sciences, University of Glasgow, Glasgow, G61 1QH, United Kingdom

^2^Public Health Scotland, Meridian Court, 5 Cadogan Street, Glasgow G2 6QE

^3^NIHR Nottingham Digestive Diseases Biomedical Research Unit, University of Nottingham, Nottingham NG7 2RD, United Kingdom

Current address and affiliations:

^a^Public Health Scotland, Meridian Court, 5 Cadogan Street, Glasgow G2 6QE

^b^School of Biodiversity, One Health & Veterinary Medicine, University of Glasgow, Garscube Campus, Bearsden Road, G61 1QH

^c^The Roslin Institute, University of Edinburgh, Easter Bush, Midlothian, EH25 9RG

*Corresponding author(s): Sema Nickbakhsh: [sema.nickbakhsh@glasgow.ac.uk](mailto:sema.nickbakhsh@glasgow.ac.uk); Emma Thomson: [emma.thomson@glasgow.ac.uk](mailto:emma.thomson@glasgow.ac.uk)

**Table S1:** Patient distributions across demographic and social factors for HCV-positive patients recruited from 48 hospital centres across England and Scotland.

| **Factor** | **Level** | **No. (%) of patients: Full data (n=3,829)** | **No. (%) of patients: Reduced data (n=2,851)*** |
| --- | --- | --- | --- |
| Age group | <46 years | 1457 (38.05) | 1076 (37.74) |
|  | 46-59 years | 1754 (45.81) | 1320 (46.3) |
|  | 60+ years | 617 (16.11) | 455 (15.96) |
|  | *Missing data* | 1 (0.03) | 0 (0) |
| Sex | Female | 1123 (29.33) | 821(28.8) |
|  | Male | 2696 (70.41) | 2030 (71.2) |
|  | *Missing data* | 10 (0.26) | 0 (0) |
| Ethnicity | White | 2935 (76.65) | 2206 (77.38) |
|  | Asian | 269 (7.03) | 198 (6.94) |
|  | Black | 67 (1.75) | 49 (1.72) |
|  | Other | 543 (14.18) | 398 (13.96) |
|  | *Missing data* | 15 (0.39) | 0 (0) |
| Country of Birth | UK | 2884 (75.32) | 2187 (76.71) |
|  | Non-UK | 893 (23.32) | 664 (23.29) |
|  | *Missing data* | 52 (1.36) | 0 (0) |
| Probable infection route | Blood/blood products | 488 (12.74) | 363 (12.73) |
|  | Injecting drug use | 2358 (61.58) | 1799 (63.1) |
|  | Other | 356 (9.30) | 270 (9.47) |
|  | Unknown | 572 (14.94) | 419 (14.7) |
|  | *Missing data* | 55 (1.44) | 0 (0) |
| History of heavy alcohol consumption | No | 2192 (57.25) | 1657 (58.12) |
|  | Yes | 1577 (41.19) | 1194 (41.88) |
|  | *Missing data* | 60 (1.57) | 0 (0) |

* Dataset used in statistical modelling analyses following the exclusion of patients with missing data.

**Table S2:** Patient distributions across HCV and co-morbidity factors for HCV-positive patients recruited from 48 hospital centres across England and Scotland.

| **Factor** | **Level** | **No. (%) of patients: Full data (n=3,829)** | **No. (%) of patients: Reduced data (n=2,851)*** |
| --- | --- | --- | --- |
| Stage of liver disease^†^ | Cirrhosis | 1385 (36.17) | 1042 (36.55) |
|  | No cirrhosis | 2444 (63.83) | 1809 (63.45) |
|  | *Missing data* | 0 (0) | 0 (0) |
| Treatment status at enrolment | Untreated | 2730 (71.30) | 1978 (69.38) |
|  | Non-DAA | 1019 (26.61) | 811 (28.45) |
|  | DAA | 71 (1.85) | 54 (1.89) |
|  | Treatment type known | 9 (0.24) | 8 (0.28) |
|  | *Missing data* | 0 (0) | 0 (0) |
| HCV genotype | 1 | 1978 (51.66) | 1576 (55.28) |
|  | 3 | 1300 (33.95) | 1033 (36.23) |
|  | Other^¥^ | 322 (8.41) | 242 (8.49) |
|  | *Missing data* | 229 (5.98) |  |
| HIV coinfection | Yes | 174 (4.54) | 137 (4.81) |
|  | No | 3428 (89.53) | 2714 (95.19) |
|  | *Missing data* | 227 (5.93) | 0 (0) |
| Body mass index (BMI) | Low (13.12 - 18.49) | 86 (2.25) | 73 (2.56) |
|  | Normal (18.5 - 25) | 1367 (35.70) | 1145 (40.16) |
|  | High (25.01 - 29.99) | 1195 (31.21) | 1023 (35.88) |
|  | Obese (30 - 60.08) | 719 (18.78) | 610 (21.4) |
|  | *Missing data* | 462 (12.07) | 0 (0) |
| Diabetes | Yes | 387 (10.11) | 296 (10.38) |
|  | No | 3415 (89.19) | 2555 (89.62) |
|  | *Missing data* | 27 (0.71) | 0 (0) |

*Dataset used in statistical modelling analyses following the exclusion of patients with missing data.

^†^Patients with cirrhosis diagnosed at any stage during the 31-month study period were assumed to be cirrhotic at enrolment

^¥^HCV genotypes 2, 4, 5, 6 and mixed genotype infections

**Table S3:** Patient distributions across health authority areas of residence for HCV-positive patients recruited from 48 hospital centres across England and Scotland.

| **Country** | **Regional Health Authorities/Boards** | **No. (%) of patients: Full data (n=3,829)** | **No. (%) of patients: Reduced data (n=2,851)*** |
| --- | --- | --- | --- |
| England | East Midlands | 477 (12.46) | 397 (13.92) |
|  | East of England | 323 (8.44) | 233 (8.17) |
|  | London | 383 (10.00) | 236 (8.28) |
|  | North East | 222 (5.80) | 182 (6.38) |
|  | North West | 284 (7.42) | 214 (7.51) |
|  | South Central | 258 (6.74) | 196 (6.87) |
|  | South East Coast | 43 (1.12) | 34 (1.19) |
|  | South West | 240 (6.27) | 123 (4.31) |
|  | West Midlands | 275 (7.18) | 225 (7.89) |
|  | Yorkshire & The Humber | 353 (9.22) | 280 (9.82) |
|  | All regions of England | 2858 (70.60) | 2120 (74.36) |
| Scotland^†^ | Ayrshire & Arran | 13 (0.34) | 9 (0.32) |
|  | Borders | 20 (0.52) | 16 (0.56) |
|  | Fife | 14 (0.37) | 14 (0.49) |
|  | Forth Valley | 21 (0.55) | 12 (0.42) |
|  | Grampian | 89 (2.32) | 81 (2.84) |
|  | Greater Glasgow & Clyde | 523 (13.66) | 363 (12.73) |
|  | Highland | 14 (0.37) | 6 (0.21) |
|  | Lanarkshire | 11 (0.29) | 10 (0.35) |
|  | Lothian | 113 (2.95) | 76 (2.67) |
|  | Tayside | 153 (4.00) | 144 (5.05) |
|  | All regions of Scotland | 971 (23.99) | 731 (25.64) |

*Dataset used in statistical modelling analyses following the exclusion of patients with missing data.

^†^Patients residing in NHS Shetland and NHS Western Isles were excluded due to small numbers. No patients were recruited from NHS Dumfries & Galloway or NHS Orkney.

**Table S4:** Prevalence (95% confidence intervals) of severe liver disease and treatment by local health authority of residence. Based on 3,829 patients attending HCV-specialist clinics in England and Scotland.

| **Health authority area** | **% Severe liver disease (95% CI)** | **% Treated pre-enrolment (95% CI)** |
| --- | --- | --- |
| East Midlands | 37.53 (33.17-42.04) | 36.06 (31.74-40.55) |
| East of England | 45.82 (40.29-51.43) | 37.05 (31.87-42.67) |
| London | 44.65 (39.6-49.78) | 24.54 (20.31-29.17) |
| North East | 28.83 (22.96-35.27) | 36.49 (30.15-43.19) |
| North West | 48.24 (42.3-54.22) | 29.93 (24.66-35.62) |
| South Central | 36.05 (30.18-42.23) | 38.37 (32.41-44.61) |
| South East Coast | 53.49 (37.65-68.82) | 37.21 (22.98-53.27) |
| South West | 37.92 (31.75-44.38) | 29.93 (24.66-35.62) |
| West Midlands | 35.64 (29.98-41.61) | 14.55 (10.60-19.28) |
| Yorkshire and The Humber | 26.91 (22.35-31.86) | 28.33 (23.69-33.34) |
| Ayrshire and Arran | 30.77 (9.09-61.43) | 61.54 (31.58-86.14) |
| Borders | 35 (15.39-59.22) | 20.0 (5.73-43.66) |
| Fife | 7.14 (0.18-33.87) | 21.43 (4.66-50.80) |
| Forth Valley | 14.29 (3.05-36.34) | 52.38 (29.78-74.29) |
| Grampian | 23.6 (15.24-33.78) | 39.33 (29.13-50.25) |
| Greater Glasgow and Clyde | 33.84 (29.79-38.08) | 21.03 (17.62-24.78) |
| Highland | 28.57 (8.39-58.1) | 28.57 (8.39-58.10) |
| Lanarkshire | 45.45 (16.75-76.62) | 45.45 (16.75-76.62) |
| Lothian | 36.28 (27.45-45.86) | 39.82 (30.73-49.46) |
| Tayside | 15.03 (9.77-21.7) | 9.15 (5.09-14.88) |
| **Measure of Geographical Variation (95% CI)^†^** | 35.45 (27.32-52.32) | 39.52 (30.46-58.33) |

^†^Coefficient of Variation (CV) with correction for sample size and 95% CI using Normal Approximation method

**Table S5**: Comparing prevalence of severe liver disease among all patients attending HCV specialist services in Scotland with the proportion with severe liver disease among the recruited study population.

| **NHS board of residence**** | **Scottish HCV Clinical database,**  **2012-2014****** | | | **HCV Research UK (HCVRUK) study population, 2012-2014** | | |
| --- | --- | --- | --- | --- | --- | --- |
|  | **Diagnosed with severe liver disease, n(%N)*** | **Total attending HCV specialist services, (N)** | **Rank from high-to-low prevalence** | **Diagnosed with severe liver disease, n(%N)*** | **Total recruited into HCVRUK over study period, (N)** | **Rank from high-to-low proportions** |
| Greater Glasgow & Clyde | 981 (24.5) | 4,010 (100.0) | 1 | 177 (33.8) | 523 (100) | 4 |
| Lothian*** | 115 (24.3) | 473 (100.0) | 2 | 41 (36.3) | 113 (100) | 2 |
| Lanarkshire | 135 (16.3) | 828 (100.0) | 3 | 5 (45.5) | 11 (100) | 1 |
| Ayrshire & Arran | 105 (14.9) | 706 (100.0) | 4 | 4 (30.8) | 13 (100) | 5 |
| Highland | 45 (13.3) | 339 (100.0) | 5 | 4 (28.6) | 14 (100) | 6 |
| Grampian | 174 (13.2) | 1,319 (100.0) | 6 | 21 (23.6) | 89 (100) | 7 |
| Borders | 6 (11.1) | 54 (100.0) | 7 | 7 (35.0) | 20 (100) | 3 |
| Tayside | 105 (10.8) | 976 (100.0) | 8 | 23 (15.0) | 153 (100) | 8 |
| Fife | 27 (9.4) | 287 (100.0) | 9 | 1 (7.1) | 14 (100) | 10 |
| Forth Valley | 29 (5.9) | 494 (100.0) | 10 | 3 (14.3) | 21 (100) | 9 |
| Total | 1,722 (18.2) | 9,486 (100.0) | - | 286 (29.5) | 971 (100) | - |

*Patients attending HCV specialist services with a diagnosis confirmed at any point up to the end of the study period (30^th^ September 2014).

**NHS Shetland and NHS Western Isles were excluded due to small numbers. No patients were recruited from NHS Dumfries & Galloway and NHS Orkney.

***Excluding Edinburgh Western General.

****Source Public Health Scotland.

Colour coding groups together regions with similar ranks across Scottish HCV Clinical Database and HCVRUK, NHS Greater Glasgow & Clyde and Borders anomalies.

**Table S6:** Prevalence (95% confidence intervals) of patient demographic and social factors by local health authority of residence. Based on 3,829 patients attending HCV-specialist clinics in England and Scotland.

| **Health authority area** | **Median age (IQR)** | **% Male (95% CI)** | **% White ethnicity (95% CI)** | **% Born in UK (95% CI)** | **% IDU route (95% CI)** | **% Blood product route (95% CI)** | **% Heavy alcohol user (95% CI)** |
| --- | --- | --- | --- | --- | --- | --- | --- |
| East Midlands | 50 (IQR=15) | 67.09 (62.67-71.29) | 71.28 (66.99-75.3) | 71.94 (67.66-75.94) | 60.34 (55.77-64.77) | 13.29 (10.37-16.68) | 40.76 (36.29-45.35) |
| East of England | 54 (IQR=13) | 69.57 (64.22-74.55) | 74.14 (68.99-78.85) | 72.5 (67.26-77.32) | 56.88 (51.25-62.37) | 18.75 (14.62-23.47) | 41.25 (35.8-46.86) |
| London | 52 (IQR=14.75) | 69.82 (64.93-74.39) | 45.62 (40.51-50.8) | 44.59 (39.46-49.82) | 42.71 (37.65-47.87) | 13.26 (10.01-17.11) | 34.7 (29.83-39.82) |
| North East | 48 (IQR=18.75) | 71.62 (65.2-77.45) | 81.45 (75.69-86.35) | 80.18 (74.32-85.21) | 56.76 (49.96-63.37) | 16.67 (12.01-22.23) | 42.73 (36.1-49.55) |
| North West | 49 (IQR=11) | 70.11 (64.38-75.4) | 89.01 (84.76-92.41) | 89.82 (85.62-93.13) | 68.79 (63.03-74.16) | 8.87 (5.82-12.81) | 47.87 (41.91-53.88) |
| South Central | 52 (IQR=13) | 72.27 (66.35-77.66) | 76.74 (71.1-81.76) | 73.64 (67.82-78.91) | 62.35 (56.09-68.32) | 13.33 (9.41-18.13) | 40.55 (34.46-46.87) |
| South East Coast | 55 (IQR=13.5) | 72.09 (56.33-84.67) | 72.09 (56.33-84.67) | 69.77 (53.87-82.82) | 59.52 (43.28-74.37) | 26.19 (13.86-42.04) | 35.71 (21.55-51.97) |
| South West | 51 (IQR=13) | 69.17 (62.9-74.95) | 92.05 (87.86-95.15) | 89.12 (84.47-92.77) | 67.36 (61.02-73.27) | 16.32 (11.87-21.63) | 49.79 (43.28-56.31) |
| West Midlands | 49 (IQR=18) | 70.07 (64.27-75.43) | 54.74 (48.64-60.74) | 54.61 (48.48-60.65) | 45.42 (39.41-51.53) | 15.38 (11.32-20.22) | 25.82 (20.75-31.42) |
| Yorkshire and The Humber | 46 (IQR=16) | 69.69 (64.6-74.44) | 73.3 (68.35-77.85) | 75.28 (70.43-79.7) | 67.06 (61.8-72.01) | 10.20 (7.21-13.91) | 42.53 (37.27-47.91) |
| Ayrshire and Arran | 54 (IQR=7) | 53.85 (25.13-80.78) | 100 (75.29-100) | 100 (75.29-100) | 25 (5.49-57.19) | 41.67 (15.17-72.33) | 30.77 (9.09-61.43) |
| Borders | 46 (IQR=15) | 85 (62.11-96.79) | 90 (68.3-98.77) | 85 (62.11-96.79) | 85 (62.11-96.79) | 10.00 (1.23-31.70) | 60 (36.05-80.88) |
| Fife | 43 (IQR=12.25) | 85.71 (57.19-98.22) | 100 (76.84-100) | 100 (76.84-100) | 71.43 (41.9-91.61) | 7.14 (0.18-33.87) | 50 (23.04-76.96) |
| Forth Valley | 53 (IQR=13) | 52.38 (29.78-74.29) | 100 (83.89-100) | 90.48 (69.62-98.83) | 63.16 (38.36-83.71) | 31.58 (12.58-56.55) | 45 (23.06-68.47) |
| Grampian | 46 (IQR=16) | 78.41 (68.35-86.47) | 87.64 (78.96-93.67) | 86.52 (77.63-92.83) | 74.16 (63.79-82.86) | 11.24 (5.52-19.69) | 51.14 (40.25-61.95) |
| Greater Glasgow and Clyde | 46 (IQR=11) | 74.76 (70.81-78.43) | 93.68 (91.24-95.61) | 93.48 (90.96-95.47) | 75.25 (71.24-78.95) | 8.51 (6.23-11.30) | 47.05 (42.64-51.49) |
| Highland | 54 (IQR=17.75) | 57.14 (28.86-82.34) | 92.86 (66.13-99.82) | 85.71 (57.19-98.22) | 30.77 (9.09-61.43) | 38.46 (13.86-68.42) | 46.15 (19.22-74.87) |
| Lanarkshire | 55 (IQR=12.5) | 54.55 (23.38-83.25) | 81.82 (48.22-97.72) | 81.82 (48.22-97.72) | 36.36 (10.93-69.21) | 54.55 (23.38-83.25) | 27.27 (6.02-60.97) |
| Lothian | 49 (IQR=14) | 70.8 (61.5-78.97) | 88.5 (81.13-93.73) | 87.5 (79.92-92.99) | 72.07 (62.76-80.17) | 7.21 (3.16-13.71) | 47.32 (37.81-56.98) |
| Tayside | 37 (IQR=16) | 71.24 (63.38-78.26) | 92.81 (87.5-96.36) | 93.46 (88.31-96.82) | 87.58 (81.29-92.36) | 3.92 (1.45-8.34) | 41.18 (33.29-49.41) |
| **Measure of Geographical Variation** | 9.33 (7.19-13.77)^†^ | 12.93 (9.96-19.08)^†^ | 17.42 (13.43-25.71)^†^  11.54 (6.44-18.51)^‡^ | 16.99 (13.10-25.08)^†^ | 27.87 (21.48-41.13)^†^ | 74.99 (57.81-110.69)^†^  36.07 (10.37-57.60)^‡^ | 19.54 (15.06-28.84)^†^ |

^†^Coefficient of Variation (CV) with correction for sample size and 95% CI using Normal Approximation method. Asymptotic test of equivalence of CV p<0.001

^‡^Coefficient of Quartile Variation (CQV) and 95% CI using Normal Approximation method; applied to variables with visually highly skewed distributions and supported by Shapiro-Wilk test of normality (p-value <0.05).

**Table S7:** Prevalence (95% confidence intervals) of patients HCV-related and comorbidity factors by local health authority of residence. Based on 3,829 patients attending HCV-specialist clinics in England and Scotland.

| **Health authority area** | **% Diabetic (95% CI)** | **% Obese (95% CI)** | **% HCV-genotype 1 (95% CI)** | **% HCV-genotype 3 (95% CI)** | **% HIV positive**  **(95% CI)** |
| --- | --- | --- | --- | --- | --- |
| East Midlands | 11.11 (8.43-14.28) | 28.15 (24.01-32.59) | 53.2 (48.49-57.87) | 37.53 (33.05-42.17) | 5.02 (3.21-7.44) |
| East of England | 13.93 (10.35-18.19) | 21.27 (16.88-26.21) | 68.77 (63.35-73.83) | 24.29 (19.67-29.4) | 2 (0.65-4.61) |
| London | 12.57 (9.41-16.31) | 17.79 (13.62-22.61) | 52.63 (47.19-58.03) | 32.46 (27.52-37.7) | 10.86 (7.84-14.55) |
| North East | 9.91 (6.32-14.62) | 22.16 (16.53-28.67) | 58.57 (51.59-65.31) | 34.29 (27.89-41.13) | 2.73 (1.01-5.84) |
| North West | 10.71 (7.35-14.94) | 26.54 (21.27-32.35) | 47.29 (41.06-53.57) | 46.12 (39.92-52.42) | 1.47 (0.4-3.71) |
| South Central | 9.73 (6.39-14.02) | 20.85 (15.58-26.96) | 59.2 (52.83-65.35) | 28 (22.53-34.01) | 2.75 (1.11-5.57) |
| South East Coast | 11.63 (3.89-25.08) | 15.38 (5.86-30.53) | 58.97 (42.1-74.43) | 38.46 (23.36-55.38) | 4.76 (0.58-16.16) |
| South West | 12.5 (8.59-17.36) | 19.31 (13.23-26.69) | 55.75 (49.02-62.34) | 31.86 (25.84-38.36) | 1.41 (0.29-4.06) |
| West Midlands | 15.3 (11.21-20.18) | 22.3 (17.47-27.76) | 45.87 (39.47-52.37) | 43.8 (37.45-50.3) | 4.04 (2.04-7.12) |
| Yorkshire and The Humber | 10.51 (7.51-14.2) | 22.44 (17.93-27.48) | 53.24 (47.78-58.64) | 39.71 (34.47-45.12) | 2.93 (1.42-5.33) |
| Ayrshire and Arran | 0 (0-24.71) | 10 (0.25-44.5) | 76.92 (46.19-94.96) | 15.38 (1.92-45.45) | 8.33 (0.21-38.48) |
| Borders | 10 (1.23-31.7) | 21.05 (6.05-45.57) | 57.89 (33.5-79.75) | 42.11 (20.25-66.5) | 27.78 (9.69-53.48) |
| Fife | 0 (0-23.16) | 7.14 (0.18-33.87) | 71.43 (41.9-91.61) | 28.57 (8.39-58.1) | 7.14 (0.18-33.87) |
| Forth Valley | 9.52 (1.17-30.38) | 5.88 (0.15-28.69) | 80.95 (58.09-94.55) | 14.29 (3.05-36.34) | 0 (0-17.65) |
| Grampian | 4.55 (1.25-11.23) | 12.64 (6.48-21.5) | 67.86 (56.78-77.64) | 25 (16.19-35.64) | 2.25 (0.27-7.88) |
| Greater Glasgow and Clyde | 5.78 (3.93-8.15) | 21.52 (17.85-25.56) | 53.11 (48.62-57.56) | 39.48 (35.16-43.92) | 3.68 (2.2-5.76) |
| Highland | 7.69 (0.19-36.03) | 0 (0-33.63) | 53.85 (25.13-80.78) | 30.77 (9.09-61.43) | 7.14 (0.18-33.87) |
| Lanarkshire | 9.09 (0.23-41.28) | 30 (6.67-65.25) | 54.55 (23.38-83.25) | 36.36 (10.93-69.21) | 0 (0-28.49) |
| Lothian | 6.6 (2.7-13.13) | 13.59 (7.63-21.75) | 65.05 (55.02-74.18) | 25.24 (17.2-34.76) | 33 (23.92-43.12) |
| Tayside | 2.61 (0.72-6.56) | 13.25 (8.28-19.71) | 37.67 (29.79-46.06) | 57.53 (49.09-65.67) | 1.96 (0.41-5.62) |
| **Measure of Geographical Variation** | 47.68 (36.75-70.37)^†^ | 44.07 (33.97-65.04)^†^ | 17.80 (13.72-26.27)^†^ | 31.0 (23.89-45.75)^†^ | 146.09 (112.61-215.62)^†^  55.56 (30.06-83.05)^‡^ |

^†^Coefficient of variation (CV) with correction for sample size and 95% CI using Normal Approximation method. Asymptotic test of equivalence of CV p<0.001

^‡^Coefficient of Quartile Variation (CQV) and 95% CI using Normal Approximation method; applied to variables with visually highly skewed distributions and supported by Shapiro-Wilk test of normality (p-value <0.05).
